# Successful pericardial repair and coverage for late pacemaker lead-related atrial perforation and pneumothorax: a case report

**DOI:** 10.1186/s44215-023-00117-3

**Published:** 2023-11-22

**Authors:** Yoshiyuki Maruya, Takumi Yamaura, Hayato Mine, Hiroyuki Suzuki

**Affiliations:** 1 Present Address: Department of Chest Surgery, Fukushima Medical University School of Medicine, Fukushima, 960-1295 Japan; 2 Present Address: Department of Chest Surgery, Takeda General Hospital, Fukushima, 965-8585 Japan

**Keywords:** Pneumothorax, Pacemaker, Myocardial perforation, Screw-in lead, Pericardial repair, Expanded polytetrafluoroethylene sheet, Video-assisted surgery

## Abstract

**Background:**

Various complications of permanent pacemaker implantation have been reported. However, late pacemaker lead-related myocardial perforation rarely occurs. Conservative treatment is generally selected, if possible, but open heart surgery or catheter lead removal should be considered in symptomatic patients. We herein describe a patient who was successfully treated by pericardial repair and coverage for late pacemaker lead-related atrial perforation and pneumothorax.

**Case presentation:**

A woman in her 80s who had undergone permanent pacemaker implantation 2 years previously visited our hospital because of dyspnea. She had also been treated for right pneumothorax 1 year previously. A chest radiograph and computed tomography scan showed right pneumothorax and pericardial emphysema with no effusion. Because similar findings had been obtained at the previous onset of pneumothorax, we suspected delayed myocardial perforation and lung injury due to the screw-in lead in the right atrium. No myocardial bleeding, cardiac tamponade, or pacing failure was present. The cardiovascular surgeon judged that open-heart lead extraction would be difficult because of the patient’s poor performance status; therefore, thoracoscopic pericardial repair with an expanded polytetrafluoroethylene sheet and coverage with anterior mediastinal adipose tissue was attempted to prevent recurrent pneumothorax. The patient was successfully treated without lead extraction or open heart surgery. At 1.5 years postoperatively, she had developed no recurrence of pneumothorax or pacing failure.

**Conclusions:**

Pericardial repair and coverage can be an effective strategy for pacemaker lead-related pneumothorax without pacing failure or bleeding.

## Background

Complications of permanent pacemaker implantation (PMI) have been reported, including myocardial perforation, pneumothorax by transvenous lung puncture, pericardial effusion, infection, and lead injury [1, 2]. In particular, late myocardial perforation due to the lead tip is a rare complication, and no standard treatment has been established. Conservative treatment may be selected in some patients whose condition is stable [3–5]. If symptoms are present, open heart surgery or catheter lead removal should be considered [6–9]. We herein report a case of pacemaker lead-related late right atrial myocardial perforation and repeated pneumothorax that was successfully treated with thoracoscopic pericardial repair with expanded polytetrafluoroethylene (ePTFE) sheets and anterior mediastinal fat tissue to avoid lead removal by open heart surgery.

## Case presentation

**Fig. 1:**
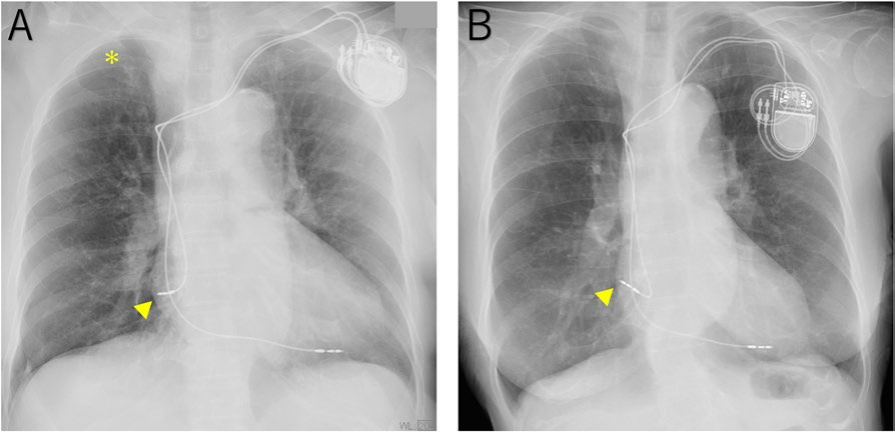
Chest X-ray images. A X-ray images taken in the sitting position on admission. The asterisk (✻) indicates the area of the collapsed lungs. The yellow arrowhead indicates the tip of the lead. **B** X-ray images were taken immediately after pacemaker implantation. The yellow arrowhead indicates the tip of the lead. The position of the tip has migrated

**Fig. 2:**
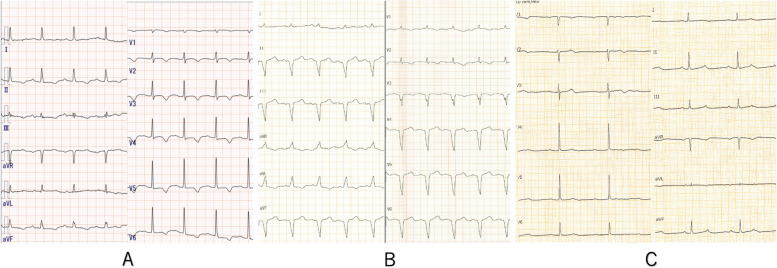
Electrocardiogram. **A** Electrocardiogram on admission shows a self-pulse. **B** Electrocardiogram after pacemaker implantation shows pacing waveforms. **C** Electrocardiogram on complete atrioventricular block

**Fig. 3:**
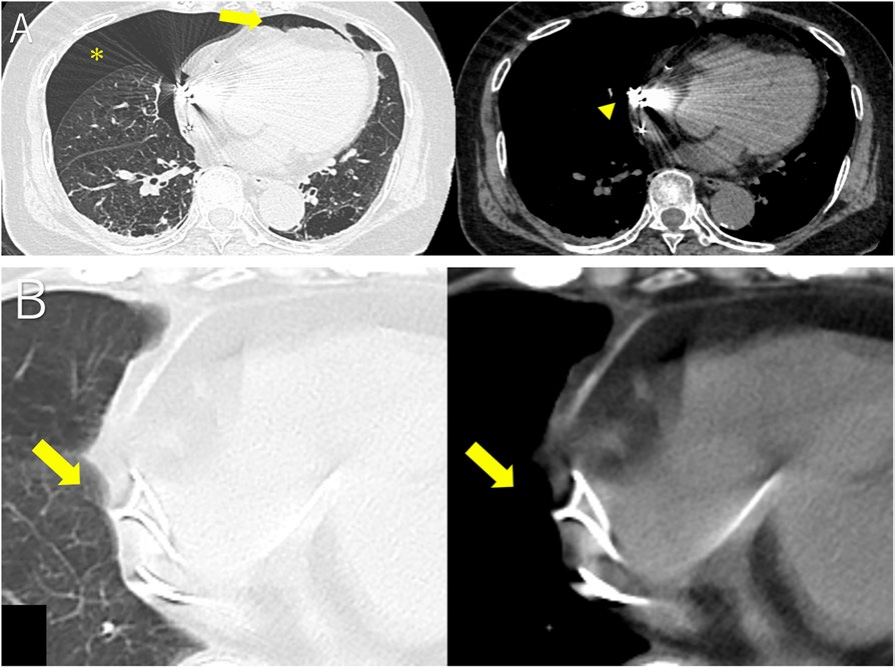
Computed tomography images. **A** The asterisk (✻) indicates the area of the collapsed lungs. The yellow arrow indicates pericardial emphysema. The yellow arrowhead indicates the tip of the lead. The tip appears to be outside the myocardial wall. **B** Postoperative computed tomography image. Pneumothorax has not recurred. The yellow arrows indicate residual adipose tissue at the tip of the right atrial lead

A woman in her early 80s presented with a 10-day history of dyspnea and chest discomfort. She had undergone PMI (dual chamber pacemaker-defibrillator with screw-in lead) for complete atrioventricular block 2 years previously, and she had developed right pneumothorax that was treated by chest drainage 1 year previously. She was an ex-smoker (61 pack-years), 142.0 cm tall, and 50.2 kg in weight, and her performance status is 1. A chest X-ray showed collapse of the right lung (Fig. 1A). Compared with the image obtained immediately after PMI (Fig. 1B), the right atrial lead tip was misaligned. An electrocardiogram showed a heart rate of 81 beats per minute and sinus rhythm with self-pulsation (Fig. 2A), whereas the electrocardiogram obtained immediately after PMI showed a pacing waveform (Fig. 2B). The pacemaker worked normally with no pacing failure. Chest computed tomography (CT) showed right pneumothorax and pericardial emphysema (Fig. 3A). No pleural or pericardial effusion was present. However, the tip of the right atrial lead appeared to be located outside the right atrial wall. Delayed myocardial perforation and repeated pneumothorax due to the right atrial lead were suspected. A treatment plan was discussed among respiratory surgeons, cardiologists, and cardiovascular surgeons, who considered that open chest surgery to remove the lead would be difficult because of the patient’s poor performance status. Fortunately, no cardiac bleeding, tamponade, or pacing failure had occurred. Therefore, a thoracoscopic covering of the lead tip was attempted.

**Fig. 4:**
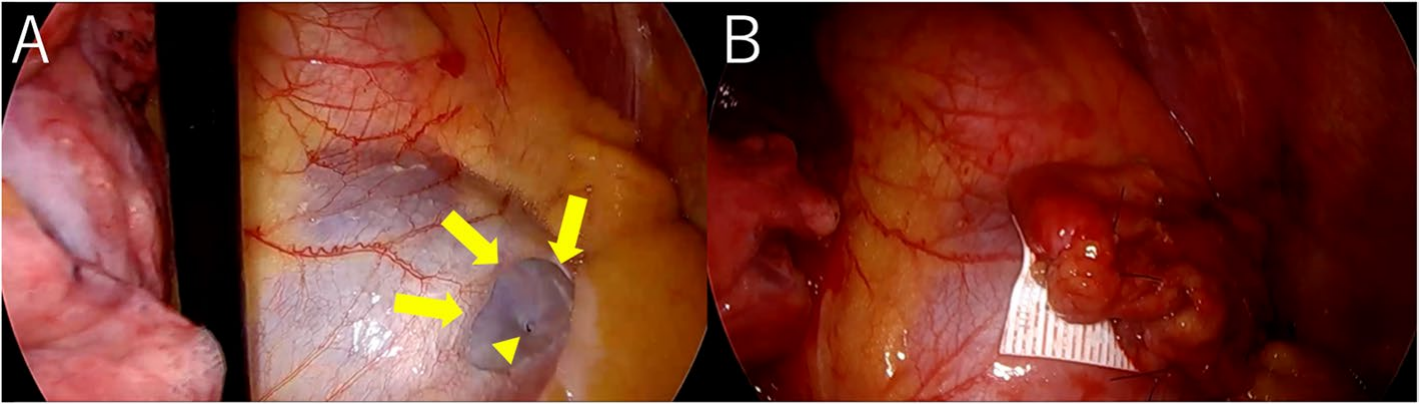
Surgical findings. **A** The yellow arrowhead indicates lead tip protrusion from the right atrial wall. The yellow arrows indicate pericardial defects. **B** An expanded polytetrafluoroethylene sheet and anterior mediastinal adipose tissue were sutured to the pericardial defect

The surgical findings are shown in Fig. 4. The pacemaker lead had perforated the right atrial wall. The pericardium at the same site was ruptured, and the defect was almost 1 × 2 cm in size. The scratched pleura of the lung that was in contact with the lead had turned white and become thickened with inflammation. The pericardial defect was repaired with an ePTFE sheet. We used the ePTFE sheet that was 2 × 3 cm in size and sutured the ePTFE sheet to the pericardium at four locations using 4-0 prolene ®(polypropylene). In addition, anterior mediastinal fat tissue made use of a pedicled fat flap and sutured over the ePTFE sheet at two locations using 4-0 prolene ®. The double-layer reinforcement was used as a pericardial replacement. The scratched lung site was reinforced using fibrin glue and polyglycolic acid felt. The patient had a good post-operative course and was discharged within a few days. At 1.5 years after surgery, the pneumothorax had not recurred and the pacemaker had not failed. Postoperative CT images indicated residual adipose tissue at the lead tip (Fig. 3B).

## Discussion and conclusions

Lead perforation reportedly occurs in approximately 0.1% to 6.0% of cases as a complication of permanent pacemaker or implantable cardioverter-defibrillator implantation [6]. Lead perforation can be classified as early perforation, which occurs within 1 month after surgery, or delayed perforation, which occurs more than 1 month after surgery; most cases are early perforation [10]. In our case, the initial pneumothorax was also considered to be due to lead perforation, which was a delayed perforation occurring 1 year after PMI. Risk factors for myocardial perforation include the use of a screw-in lead, advanced age, a small build, and corticosteroid use [3]. Our patient had three of these risk factors: use of a screw-in lead, advanced age, and a small build. Another factor contributing to the perforation was that the lead, which had been implanted in the right auricle, had migrated to the free wall of the right atrium. According to previous reports, the length of the screw-in lead is 1.6 to 1.8 mm and the atrial wall is only about 2 mm; thus, there is a possibility of perforation when the tip vertically contacts the atrial wall due to positional change [2, 5]. A CT scan is considered standard for the diagnosis of lead perforation [7], and we suspected myocardial perforation by the lead because the lead tip had protruded outside the right atrial wall on the CT scan. However, a CT scan may not be able to accurately depict the lead tip because of strong artifacts. Catheter angiography may be considered as an additional test when the CT findings are unclear [8]. Myocardial perforation by the lead can cause a variety of symptoms, including pneumothorax, hemothorax, cardiac tamponade, diaphragmatic injury, and pericarditis. However, many patients are reportedly asymptomatic or show nonspecific symptoms [10]. In a previous report of 111 autopsy cases with PMI, myocardial perforation or penetration was found in 7 (6.3%) cases and atrial perforation in 3 (2.7%). Notably, the three patients with atrial perforation had no pacing failure [11]. Likewise, our patient had no hemorrhage or pacing failure; therefore, the myocardial perforation was presumed to have gradually developed.

A perforated lead causes traffic between the thoracic and pericardial cavities and lung injury, leading to pneumothorax and pneumopericardium. Routine imaging evaluation is considered necessary even in the absence of pacing failure. There is no established treatment for lead perforation. Several reports have described improvement with observation only (no lead removal) [3–5]. However, if lead perforation causes uncontrolled hemorrhage or lung injury, lead removal is unavoidable. Lead removal can be performed by transvenous or open-heart lead extraction. In a report of 51 patients with delayed lead perforation, 11 patients underwent transvenous extraction and 14 underwent open-heart lead extraction [10]. Transvenous removal carries a risk of bleeding and should be performed in the operating room in the presence of a cardiovascular surgeon. Other reports have described cases in which lead removal was performed with monitoring of bleeding by fluoroscopy and trans-esophageal echocardiography [9]. Open-heart lead removal is the most reliable technique because it can be performed while checking for bleeding, but it has the disadvantage of being highly invasive. In this case, the patient developed repeated pneumothorax; therefore, catheter removal was first considered a conservative treatment. However, because pacing failure had not occurred and the patient had a poor performance status, making her unlikely to tolerate bleeding or open heart surgery, thoracoscopic pericardial repair was chosen. An ePTFE sheet was sutured to the perforation site as a substitute pericardium, and the fat tissue of the anterior mediastinum made use of a pedicled fat flap and sutured over the ePTFE sheet. This technique was relatively easy to perform and was not highly invasive. Although no previous reports have described this technique, the patient had not developed a recurrence of pneumothorax at 1.5 years postoperatively. This may be an effective treatment strategy when lead removal by open heart surgery is not possible.

We will follow-up regularly to check for complications such as recurrent pneumothorax, bleeding, and infection. If our patient gets a pneumothorax again or becomes infected, we must consider removing the lead.

## Data Availability

Not applicable.

## Abbreviations

PMI: Pacemaker implantation
CT: Computed tomography
ePTFE: Expanded polytetrafluoroethylene
